# The Role of Bispecific Antibodies in Non-Hodgkin’s Lymphoma: From Structure to Prospective Clinical Use

**DOI:** 10.3390/antib11010016

**Published:** 2022-02-21

**Authors:** Rita Tavarozzi, Enrica Manzato

**Affiliations:** 1 Department of Translational Medicine, University of Eastern Piedmont, 28100 Novara, Italy; 2 SCDU of Hematology, Azienda Ospedaliera SS Antonio e Biagio e Cesare Arrigo, 15121 Alessandria, Italy; 3 Institute of Life Sciences, Sant’Anna School of Advanced Studies, 56127 Pisa, Italy; enrica.manzato@gmail.com

**Keywords:** bispecific antibody, bispecific T-cell engager, non-Hodgkin’s lymphoma, blinatumomab

## Abstract

Bispecific antibodies (bsAbs) are molecules that simultaneously bind two different antigens (Ags). bsAbs represent a very active field in tumor immunotherapy with more than one hundred molecules currently being tested. More specifically, they have elicited a great interest in the setting of non-Hodgkin’s lymphoma (NHLs), where they could represent a viable option for more fragile patients or those resistant to other conventional therapies. This review aims to give a brief overview of the different available bsAb formats and their mechanisms of action, pinpointing the differences between IgG-like and non-IgG-like classes and will then focus on those in advanced clinical development for NHLs.

## 1. Introduction

Bispecific antibodies (bsAbs) are antibodies (Abs) or Ab-derived structures that exhibit two different binding sites, and thus can specifically link two different antigens (Ags) [1]. Their history started back in the 1960s, when initial attempts demonstrated how Abs presenting two identical Ag binding domains could be combined [2,3,4]. However, it was only afterwords, due to the refinement of different approaches, that they were introduced into clinical scenarios [5,6]. In addition, many efforts focused on improving immunological platforms were made [7,8,9,10,11,12] increasing feasibility and efficacy of these products. As a result, the Federal Drug Administration (FDA) has approved the first-in-class bsAb, blinatumomab, for B-cell acute lymphoblastic leukemia (ALL) in 2014 [13]. At the moment, blinatumomab has been joined by emicizumab for hemophilia A [14] and more recently by amivantamab [15] for non-small cell lung cancer, while catumaxomab, initially approved for the treatment of malignant ascites, has been withdrawn from the market in the European Union [16]. Actually, there are over one hundred bsAbs currently being tested making this an active and rapidly expanding field where it can be difficult to keep track of all the newest developments.

Blinatumomab, the first bsAbs approved for clinical indication, spurred a great interest in the use of bsAbs in relapse/refractory (R/R) lymphoproliferative disorders [17]. More specifically, there has been a certain appeal in in use of bsAbs in relapsed non-Hodgkin’s lymphomas (NHLs) where immunotherapy approaches have already been successful [18,19].

The following sections highlight the characterization and mechanism of action of bsAbs approved or in clinical development for NHLs. In particular, we describe the leading clinical agents (i.e., blinatumomab, glofitamab, mosunetuzumab, odronextamab, and epcoritamab) and provide a review of the most relevant trials.

### 1.1. Formats of bsAbs: A Broad Overview

Over decades of research on the pharmacology of bsAbs, with continuous efforts towards improving their properties, two different formats have been introduced in clinical use: on one hand there are single chain fragment variable (scFv)-based Abs, which present no no fragment crystallizable (Fc) and are also known as “non-IgG-like”; on the other hand, there are bsAbs based on the full length IgG molecule, also known as “IgG-like.

**Figure 1:**
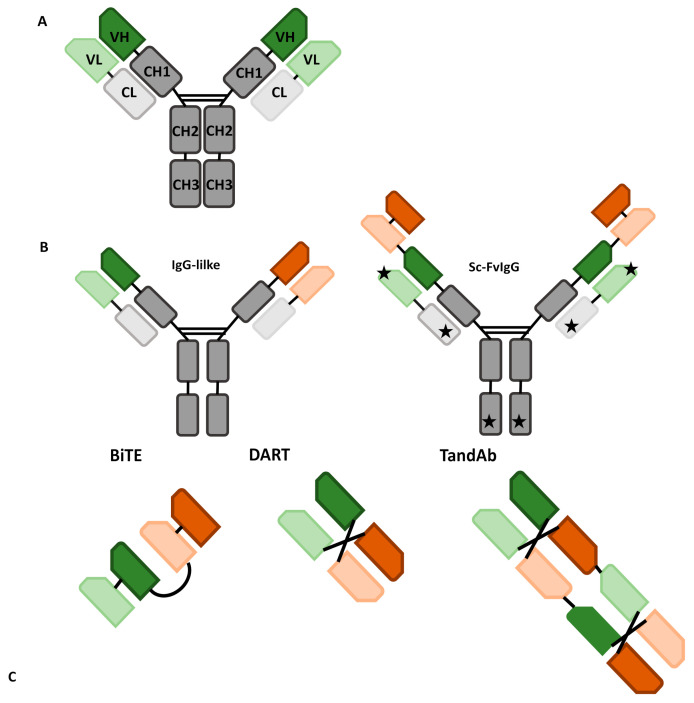
Examples of different antibodies: green and red are used for different specificities, while gray for the constant chains; and lighter colors indicate light chains, darker ones heavy chains. (**A**) Structure of a native antibody; CH: constant heavy chain, CL: constant light chain, VH: variable heavy chain, VL: variable light chain. (**B**) IgG-like antibodies; Sc-FvIgG: IgG-like antibody with added specificities through single chain fragment variables (stars indicate other sites which the ScFv can be bound to). (**C**) Non-IgG-like antibodies; BiTE: bispecific T-cell engager, DART: dual-affinity retargeting antibody, TandAb: tandem diabody.

BaAbs in the first class are characterized by the absence of a Fc-portion which influences their molecule profile. For instance, this facilitates their output. In addition, these molecules do not exert Fc-mediated effector functions such as antibody-dependent cell-mediated cytotoxicity (ADCC), antibody-dependent cellular phagocytosis (ADCP), complement fixation, and exhibit faster clearance due to the lack of a neonatal Fc receptor (FcRn) binding site which is responsible for the long half-life of most γ immunoglobulins. Non-IgG-like products include a wide variety of different formats, such as bispecific T or killer-cell engagers (BiTEs or BiKEs), dual-affinity re-targeting antibodies (DARTs) and tandem diabodies (TandAbs), all depicted in Figure 1.

In contrast, IgG-like bsAbs are derived by pairing two polypeptide chains and have the characteristics of Fc-domain-based antibodies.

### 1.2. IgG-Like bsAbs

IgG-like bsAbs exhibit very unusual and intricate biological structures, which drive their clinical properties and efficacy. All the IgG-like bsAbs resemble wild-type immunoglobulins in their primary composition. However, in the heavy- and light-chain coupling processes they may acquire an asymmetric geometry due to the presence of several Fv regions of their respective chains. Indeed, planning how to assemble the binding sites between heavy and light chains has historically been one of the most difficult tasks in the development of bsAbs.

To overcome these chemical and pharmacological issues, a variety of approaches have been tested, such as quadroma technology (now mostly outdated thanks to newer technologies), knobs-into-holes, common heavy-chain and common light-chain strategies, and CrossMab technology. Finally, genetic modifications and proteomic implementations have been introduced to optimize their production and efficacy [20,21,22,23,24,25,26,27,28,29,30,31]. 

Modifications of the heavy chains or light chains are often used to induce the correct pairing of two different modified heavy chains, or of two different light chains to the same extended heavy chain, and thus create a bispecific molecule. For instance, the knob-and-hole method of bsAbs production relies on the introduction of complementary mutations in the common heavy 3 (CH_3_) domains of two semi-Abs to promote heterodimerization and the creation of a bsAbs [20,23,31]. Knobs-into-holes heterodimerization allows proper heterodimeric coupling and also ensures that the resulting bsAbs have a stable structure [31]. Common light-chain and CrossMab technology are instead approaches acting on different fragments, such as the Fab segment. The CrossMab technology makes it possible to create various bsAbs (including bi-, tri-, and tetra-valent Abs) by exchanging the sequences of the heavy- and light-chain domains of the Fab fragments [20,23,31]. 

Besides these intricate engineering tasks, newer manufacturing technologies have stimulated the development of novel Abs through less complex changes from their primary design. New Abs with a backbone different from the IgG1 moiety, such as IgG2 [24], IgG4, ref. [25] or IgM structure, which can provide more binding sites and influence the properties of the Abs [26] are also being tested with encouraging preliminary data. 

The presence of an Fc region influences the pharmacological profile (half-life) of IgG like antibodies, including bsAbs. Indeed, binding through the Fc prevents IgG catabolism, ensuring a longer half-life of these bsAbs [20,21]. This happens because the FcRn, encoded by the fcgrt gene, is a major histocompatibility complex (MHC) class I-like molecule which naturally protects IgG from catabolism by mediating the bidirectional transcytosis of IgG through epithelial cells and membrane recycling. The FcRn-IgG interaction also works on Ag presentation and cross-presentation in Ag-presenting cells (such as macrophages and dendritic cells). Each of these functions has important implications in the development of therapeutic Abs. Indeed, various pharmacological strategies have co-opted FcRn functions by blocking the FcRn-IgG binding to facilitate endogenous IgG degradation or by modifying the Fc to facilitate the FcRn binding and extend the circulating half-life of IgGs [21]. 

In addition, the inclusion of small molecules such as aptamers, a-bodies, and synthetic drugs may further expand their applicability. For instance, one could envision a fusion protein composed of the effector domain of a macromolecule bound to the Fc region of the IgG, which would confer a prolonged half-life and improved production due to both its interactions with the FcRn and its relative stability. 

Furthermore, the constant regions of the heavy chains (CH_3_ and CH_2_) may be engineered to form novel Ag-binding sites. These new, small formats of Fc Ag-binding Abs (Fcab) have been shown to possess not only similar properties to full-size Abs, but also the added advantage of lower manufacturing costs. To further reduce the size of possible scaffolds, the Fc region of the IgG can be divided into two CH_2_ regions and two CH_3_ domains. These domains have the potential to be engineered into even smaller Ab fragments [22].

Finally, one can obtain a different number of Fab regions on different Abs by adding more Ag-binding units to either the amino- or carboxy-terminus of the light and/or heavy chains of monospecific Abs through short linkers. These Ag-binding units can consist in scFvs, unpaired variable light or heavy chains, or other protein constructs; and they can have the same or different structures, increasing valency or widening the range of specificities [27,28].

### 1.3. Non-IgG-Like bsAbs

Non-IgG-like bsAbs are functional fragments of an Ab created by fusing different elements of the wild-type immunoglobulin. The most commonly used in clinical practice, derived from the fusion of the heavy- and light-chain variable domains (VH and VL) of the IgG through a flexible polypeptide linker (i.e., scFvs). They are usually small, which lends them a greater tissue penetration but a shorter half-life (although it can be increased, at the cost of a higher toxicity, for example by binding these molecules to albumin [17]). In addition, they do not suffer from the toxicities due to the Fc region [20,29,30]. 

Currently, there are a few main bsAbs fragment formats: BiTEs, BiKes, DARTs, and TandAbs. 

BiTE molecules have been widely applied in cancer immunotherapy. They employ scFv fragments of two different monoclonal Abs connected by a peptide linker, allowing them to maintain the binding activity of each Ab when assembled [31]. This makes them able to direct the cellular T response against the target Ag. The short flexible linker connecting the two scFv allows free rotation of the two arms, which is vital for flexible interaction with targeted receptors on two opposite cell membranes (cytotoxic T-cell and tumor cell) and subsequent induction of T-cell activation [31]. This results in a small structure that on one hand can bring tumor and effector cell in close proximity but on the other it is responsible for the high clearance observed, presumably through the kidney [27,31]. 

BiKEs have a similar structure to BiTEs, but instead bind to natural killer (NK) cells. A further variation of this theme is represented by trispecific killer cell engagers (TriKEs), which incorporate interleukin-15 in their design to better activate these cells [32].

DARTs consist of two scFv fragments that are connected by a disulfide bond to give two different binding sites to Ag. Specifically, one fragment (Fv1) consists of a VH from Ab A and a VL from Ab B, while the second fragment (Fv2) is made from a VH from Ab B and VL from Ab A. This structure allows DARTs to mimic the natural interaction within an IgG molecule [31]. This feature makes them different from BiTEs, compared to whom they seem to be more active in vivo and have less production issues [33,34]. 

Despite their advantages, some limitations exist. For instance, their small size and the absence of an FcRn binding site contribute to a high rate of renal clearance compared to natural Abs. For example, blinatumomab presents a short elimination half-life (mean ± SD) of 1.25 ± 0.63 h, with predominantly renal elimination [31]. As a result, blinatumomab requires continuous, high concentration (15–28 µg per day) dosing to achieve target T-cell destruction. Therefore, this antibody is administered as a continuous intravenous (IV) infusion for 4 weeks to maintain a sufficient therapeutic serum concentration, with increased costs [31]. 

Currently, DART and BiTE proteins can be further engineered to improve their interaction with patients’ immune systems and serum half-life. For example, novel BiTE molecules can be linked to the IgG Fc domain to generate BiTE-Fc fusion drugs which are efficient with a once weekly dosing, thus extending the drug half-life and therapeutic compliance [31].

In order to extend serum half-life, TandAbs protein has been generated. These bispecific tetravalent Abs provide two binding sites for each Ag to maintain the avidity of a bivalent natural Ab. In addition, TandAbs have a molecular weight (approximately 105 kDa) that exceeds the first-pass renal clearance threshold, thus offering a longer half-life than smaller Ab constructs [31]. However, they are still smaller than IgG-like bsAbs, so still have a good tissue penetration [35]. 

Two interesting TandAb format drugs are under development. AFM13 (binding CD30 and CD16) for NK cell recruitment has showed favorable profile in clinical trials. In contrast, AFM11 (binding CD19 and CD3) for T-cell recruitment has provide discordant results in clinical trial and is suspended at the moment (NCT02848911) [36,37]. 

## 2. Mechanism of Action

BsAbs act, by definition, by binding two different Ags. A great variety of different consequences can arise: For instance, the two bound Ags can be found on the same or (more common) on different cells, and the presence or absence of the Fc region can change the Ab’s action and properties. Most bsAbs in use or in clinical trials in NHLs are either IgG-like bsAbs (mosunetuzumab, odronextamab, epcoritamab) or BiTEs (blinatumomab), so this section focuses on these two kinds; however, there are a great variety of formats and mechanisms of action being tested in early phase trials [38].

IgG-like bsAbs, as stated above, are characterized by the presence of the Fc region which determines many of their unique properties. Indeed, bsAbs with a functional Fc region are considered trifunctional Abs, or Triomabs, since they can bind the C1q to activate complement and the Fc-γ receptors on NK cells, macrophages, or dendritic cells to elicit Ab-dependent cytotoxicity and phagocytosis [17,30]. These functions can also be enhanced through careful engineering of the molecule: For instance, introducing a point mutation in the CH_2_ domain enhances binding to the Fc-γ receptor IIb and makes the Ab more effective [39]. However, this comes at the expense of a higher toxicity, especially cytokine release syndrome, as well as a risk of unwanted lysis of T-cells and more difficult formation of cytolytic synapses [30]. To avoid this, in newer bsAbs the Fc region is modified by using different IgG heavy chains and/or introducing specific point mutations in them (e.g., in the hinge region) in order to completely abolish binding to C1q and Fc-γ receptors, while still preserving FcRn binding to maintain the longer half-life. For example, mosunetuzumab benefits from CH mutations that limit its effector functions, while epcoritamab from others that abolish them completely [40]. In this manner, the therapeutic effect is mediated exclusively by the Fab arms, which in these Abs usually bind on one hand the CD3 to recruit cytotoxic T-cells and on the other a tumor Ag, which in NHLs is usually selected to be CD20 [20]. This concept is taken even further by BiTEs, which do away with the Fc region altogether and are designed to only bind the CD3 and the tumor Ag, such as CD19 in the case of blinatumomab and CD20 in the case of glofitamab. Once the bsAb has bound the CD3, the T-cell receptor complex triggers activation of the T-cell with polyclonal activation and proliferation, and formation of a cytolytic synapse with the adjacent tumor cell, which is then destroyed through the release of cytolytic granules and cytokines in the synapse [41].

## 3. Bispecific Antibodies in Use and in Development in NHL

Notwithstanding the great variety of bsAbs in preclinical development, at the moment only a few of them, summarized in Table 1, have reached advanced clinical testing. In the following sections, they are better described.

### 3.1. Blinatumomab

Blinatumomab, an anti-CD19/CD3 bsAb, was the first-in-class that showed high efficacy in the R/R setting of B-ALL and was approved by the FDA for this indication in 2014 [48,49]. 

In B-cell lymphoma malignancies, blinatumomab was been assessed as a salvage strategy in R/R NHLs [42,43,50]. In a phase I study, blinatumomab was used as a continuous infusion (for four to eight weeks, plus an additional four weeks if clinical benefit was achieved) with an escalated doses schedule. Seventy-six R/R B-NHLs (including 14 diffuse large B-cell lymphoma, DLBCL) were enrolled. Results showed that doses below 60 µg/m^2^/day were associated with poor response rates and a dose of 90 µg/m^2^/day was limited by neurotoxicity. In the extension phase, 11 patients with R/R DLBCL received the target dose with an overall response rate (ORR) of 55% and a complete remission (CR)/unconfirmed complete remission rate of 36% [42]. In a phase 1 study, neurologic events occurred in 71% of patients with >20% of them reported grade 3. No grade 4 or 5 events occurred [42]. 

Viardot et al. in a limited phase II study (21 evaluable patients) showed an ORR of 42% with a CR rate of 19% [43]. Notably, grade 3 neurologic events were reported in 22 (95.7%) of patients with encephalopathy (9%) and aphasia (9%) the most common. No patient had a grade 4 or grade 5 neurologic event and no cytokine release syndrome (CRS) was reported [43].

A phase II study by Coyle et al. evaluated blinatumomab as a second salvage in R/R DLBCL [50]. In 41 patients, after 12 weeks of therapy, the ORR was 37% with a CR rate of 22% [50]. A high rate of treatment discontinuation was reported resulting in only 59% of patients receiving more than 80% of their intended dose [50]. 

### 3.2. Glofitamab

Glofitamab is a humanized mouse-derived IgG1-like T-cell engaging bsAb possessing a novel 2:1 structure with bivalency for CD20 and monovalency for CD3. The Fc structure is characterized by the absence of FcγR and complement binding site. The presence of 2 CD20-binding sites (derived from type II CD20 IgG1 gly-coengineered Obinutuzumab) improves affinity for CD20+ target cells. 

Bacac et al. in a preclinical study had shown its superior potency compared with other tested bsAbs [51] providing novel insight to the bispecific era.

In a phase I/Ib trial glofitamab was used as single-agent in R/R B-NHLs. The monoclonal Ab CD20 obinutuzumab was administered before initiating glofitamab in order to prevent CRS, by occupying surface CD20 on the lymphoma cells and depleting peripheral B-cells. Glofitamab was given as IV infusion, in 14- or 21-day cycles for up to 12 cycles, and an escalating doses schedule of 0.6 to 25 mg was used. One hundred and seventy-one patients were enrolled with a median age of 64 (range, 22–85) years. Aggressive NHLs (aNHLs) (DLBCL, transformed follicular lymphoma (tfFL), primary mediastinal large B-cell lymphoma, PMBCL, mantle cell lymphoma, MCL, and Richter’s transformation) and indolent NHLs (iNHLs) (grade 1–3A FL) were enrolled; patients had a median of 3 (range, 1–13) prior lines of therapy and 90.6% were refractory to all prior therapy. Clinical activity was observed at all doses. Among patients with aggressive B-NHLs, ORR, and CR were 48.0% and 33.1%, respectively, including 41.1% and 28.8% in patients with DLBCL and 55.2% and 34.5% in patients with transformed FL. In grade 1-3A FL, 70.5% achieved response with high rate of CR (47.7%) [44].

In an aggressive NHLs setting, the median duration of response (DOR) was 5.5 months (95% CI, 4.4 to not estimable; range, 0.8–28.8 months) and the median progression free survival (PFS) was 2.9 (95% CI, 2.1 to 3.9) months. In grade 1–3A FL, the median DOR was 10.8 months (95% CI, 3.8 to not estimable) and the median PFS was 11.8 months (95% CI, 6.3 to 24.2) [44].

Adverse events (AEs) were reported in 98.2% of patients. The most common AE was CRS, occurring in 50.3% with grade 1 and grade 4 in 1.2% of patients. Symptoms of immune effector cell-associated neurotoxicity syndrome (ICANS) during CRS were uncommon and all resolved within 3–72 h. Incidence of CRS increased with dose but declined considerably after the first administration. Grade ≥ 3 neutropenia occurred in 25.1% of patients. Infections and febrile neutropenia occurred in 51.5% and 2.9% of patients, respectively [44].

These pieces of data were confirmed by another trial. Interim data for NP30179 presented the results of 52 patients with R/R NHLs who received glofitamab step-up dosing with obinutuzumab pretreatment to reduce toxicity [52]. Glofitamab was then administered IV in a weekly step-up dosing regimen with a schedule of either 2.5/10/16 mg or 2.5/10/30 mg. Glofitamab was given every three weeks for up to 12 cycles. A total of 52 patients were treated in the two cohorts; these patients had a mean age of 68 years (range, 44–85) and 53.8% were male. More than half of the patients (53.8%) had aNHLs (DLBCL, transformed FL, Richter’s transformation, MCL). A total of 46.2% of patients had indolent G1-3A FL. Patients were highly pretreated (median of 3 lines of therapy consisting of chemoimmunotherapy, autologous stem cell transplantation in 21.2%, a PI3K inhibitor in 9.6%, chimeric antigen receptor, CAR, T-cell therapy in 5.8%, and cancer immunotherapy in 1.9%). The ORR was 63.5% for all patients with a complete metabolic response (CMR) observed in 53.8% of patients [52].

In those with iNHLs the ORR was 66.7% and the CMR rate was 54.2% versus 60.7% and 53.6%, respectively, in the aNHLs group. Complete responses (CRs) were generally achieved early and observed from the first or second response assessment [52]. 

Almost all patients (98.1%) experienced at least 1 AE, and 88.5% had treatment-related events. No fatal AEs were reported in the study. The most common AEs related to Glofitamab treatment were CRS, neutropenia, pyrexia, and thrombocytopenia [52].

Several trials are still ongoing, and results are pending (Table 2).

### 3.3. Mosunetuzumab

Mosunetuzumab is a fully humanized bispecific IgG1 monoclonal Ab, capable of recognizing and binding the CD20 Ag as a tumor target, and the CD3 Ag on T-cells. Modified Fc is characterized by the absence of FcγR and complement binding, with only 1 binding site to CD20. 

Mosunetuzumab is now under study as monotherapy or combined with other drugs to treat B-cell NHLs.

The phase I/Ib GO29781 study (NCT02500407) evaluated mosunetuzumab monotherapy in patients with NHLs R/R (aggressive and indolent forms) [45]. Mosunetuzumab was given with a step-up dose on days 1, 8, and 15 of cycle 1, followed by a fixed dose on day 1 of each subsequent cycle up to a maximum of 17 cycles. The schedule ranged from a weekly step-up dose of 0.4/1/2.8 to 1/2/40.5 mg. Two hundred and seventy patients were evaluated (66.7% had an aNHLs—including DLBCL, tfFL, and MCL—and 31.5% an iNHLs). Of note, patients were heavily pretreated and 11.1% had received prior CAR T-cell therapy. Efficacy analysis showed high response rates in both aNHLs and iNHLs. In fact, an ORR of 37.4% (CR 19.5%) and 62.7% (CR 43.3%) was reported in aNHLs and iNHLs, respectively [45]. In CAR T-cell R/R patients the ORR was 38.9% (CR rate of 22.2%) [45]. Similarly, high response rates were reported in the POD24 population [53]. In all groups, responses were durable over months with a median DOR of 20.4 months (95% CI: 9.4–22.7) [53]. AEs were reported in 60 pts (97%); 14 pts (23%) experienced CRS. CRS events were reversible, mostly of grade < 2 and predominantly occurred during the first cycle. No patient required tocilizumab, intensive care unit admission or use of vasopressors for CRS management. Neurologic AEs (NAEs) were observed in 28 pts (45%) with headache (24%), insomnia (15%), and dizziness (11%) the most reported. No grade ≥ 3 NAEs or serious NAEs were reported [53]. These safety data are confirmed in a heavily pre-treated, R/R CAR-T cell therapies population [45]. 

A subcutaneous (SC) monotherapy with mosunetuzumab was tested in study GO29781 (NCT02500407) to investigate alternative dosing strategies. R/R patients with aNHLs or iNHLs were included, and doses of 1.6 to 20 mg oce every three weeks were considered. High efficacy rates were reported, with an ORR of 86% and a CR rate of 29% in patients with iNHLs and 60% and 20% in patients with aNHLs, respectively [54]. All responses were durable with a median of 6.9 months [54]. The SC administration of mosunetuzumab has shown a low absorption rate and high bioavailability (>75%) confirming a favorable toxicity profile compared to the IV formulation (reduced rate of grade ≥ 2 CRS at doses below 13.5 mg) [54].

In the first-line setting, the drug mosunetuzumab is being assessed as monotherapy or in combination with chemotherapy. In phase I/II study GO40554 (NCT03677154), mosunetuzumab monotherapy is being evaluated in patients 80 years of age or older or in patients with untreated DLBCL aged 60–79 years who are ineligible for R-CHOP chemotherapy. Of the 19 evaluable patients, eight patients received the weekly step-up dose of 1/2/13.5 mg while 11 patients received the 1/2/30 mg dose. Treatment was continued for up to a maximum of 17 cycles. The median age was 84 (range: 67–100) years. The ORR was 58% and the CR rate was 42% [55]. Similar efficacy data were shown by the phase Ib/II GO40515 trial (NCT03677141), in which mosunetuzumab was also evaluated in early-stage DLBCL, CHOP chemotherapy eligible. Mosunetuzumab was administered in a weekly step-up dosing regimen at a 1/2/13.5 or 1/2/30 mg dose level in R/R NHL. In the seven patients with R/R NHL, the ORR was 86% with a CR rate of 71% [56]. In the 27 evaluable DLBCL patients, the ORR was 96% and the CR rate was 85% [56]. Grade ≥ 3 AEs occurred in 37 pts (86%). Nineteen patients (53%) with previously untreated DLBCL had mild CRS events. No ICANS events were observed [56].

### 3.4. Odronextamab

Odronextamab (REGN1979) is a fully human IgG4-based CD20/CD3 bsAb, hinge-stabilized, which has been evaluated in the NHLs R/R setting. In a phase I study (NCT02290951), odronextamab was administered in a step-up dosing regimen over three weeks followed by a fixed weekly dose until week 12. After this, maintenance dosing was given. Preliminary data is available for 127 patients with R/R NHLs with doses ranging from 0.03 to 320 mg [46]. This cohort included a heavily pre-treated group of patients with DLBCL, grade 1–3a FL and MCL, with 29 patients having received prior CAR T-cell therapy. In patients with R/R grade 1–3a FL, odronextamab, at a dose of ≥5 mg, achieved an ORR of 92.9% and a CR rate of 75.0% [46]. The median DOR was 7.7 months [46]. In patients with DLBCL, excluding those who had received prior CAR T-cell therapy, in those treated at doses ≥ 80 mg (n = 10) the ORR and CR rate were both 60% [46]. The median observed DOR in the DLBCL group was 10.3 months [46]. In those DLBCL patients who relapsed after CAR T-cell therapy, 21 patients were treated at doses ≥ 80 mg, with an ORR of 33.3% and a CR rate of 23.8% [46]. Grade > 3 CRS occurred in 9 pts (7.1%) during the first 2 weeks of step-up dosing and resolved within a median of 2 days (range 1–41) with supportive care measures. No patient discontinued odronextamab treatment due to CRS. Grade 3 neurologic AEs were noted in 3 (2.3%) of patients. None of these events required treatment discontinuation. No grade 4 or higher neurologic AEs were reported [46]. 

### 3.5. Epcoritamab

Epcoritamab (GEN3013) is a CD20/CD3 IgG1 bsAb administered via SC injections [47]. In the phase I/II study (NCT03625037), patients with R/R NHLs received a SC injection of epcoritamab at a fixed weekly dose for two 28-day cycles, fortnightly for four cycles, and every four weeks thereafter. Preliminary data presented results for 67 patients (67% with DLBCL, 18% with FL, and 6% with MCL) with an ORR of 66.7% (CR rate of 33.3%) for doses > 12 mg. Notably, of 9% who had received previous CAR T-cell therapy, all responded (two patients achieving a CR and the other two a partial response) [47]. Of the seven patients with DLBCL who received a dose ≥ 48 mg, the ORR was 100% (28.6% CRs) [47]. High efficacy was also shown in FL patients who received a dose ≥ 0.76 mg with an ORR of 100% (two patients achieved a CR) [47]. In the four patients with MCL, responses were observed in two patients with blastoid variant MCL [47]. Data on the duration of response are not yet available. CRS events were all grade 1/2 (58%) with no grade 3/4 CRS events, and limited neurotoxicity was observed (6%; grade 1: 3%; grade 3: 3%; all transient). There were no dose-limiting toxicities [47].

### 3.6. Novel Perspective and bsAbs in Clinical Development

A critical challenge is the production of bsAbs with high clinical performance and reduced side effects. Looking for new immunological targets and pathways plays a leading role. 

ROR1 is a highly attractive candidate for targeted cancer therapy. ROR1 is a receptor tyrosine kinase uniformly expressed on the cell surface of malignant B cells, carcinoma, sarcoma, and melanoma solid tumors [57]. NVG-111 is a humanized, tandem scFv bsAb binding ROR1 and CD3 and previously shown to be a potent tumor cell killer in vitro. In vivo it has also been shown to engage a ROR1 membrane-proximal epitope in the Wnt5a-binding Frizzled domain and redirect T-cell activity [58]. An ongoing phase 1/2 study in patients with R/R chronic lymphocytic leukemia (CLL) and MCL is evaluating an escalated doses schedule (of 0.3 to 360 µg/day) via continuous infusion over 3 cycles (each 21 days on, 7 days off). Patients are ≥2nd line therapy with a Bruton’s tyrosine kinase inhibitor, or venetoclax (clinical trial information: 2020-000820-20). Results are pending [58]. 

CD22 is an attractive target for B-cell malignancies due to its strong expression on B-lymphoid cell surface. Several CD22-targeting monoclonal Abs, Ab-drug conjugates, radioimmunoconjugates, CAR T-cells, and bsAbs are under investigations. NCT04540796 is an ongoing phase I trial with JNJ-75348780, a bsAb targeting CD3 and CD22 in patients with R/R NHLs, including CLL. Preliminary results have not been published at this time [59]. 

Immune checkpoint inhibitors have shown high clinical activity in many tumor types; however, only a fraction of patients benefit [60]. In a preclinical setting, combining CD137 agonists with these inhibitors increased antitumor activity, but attempts to translate these observations into the clinic were hampered by systemic toxicity [61]. A bispecific human CD137x programmed death ligand 1 (PD-L1) Ab, MCLA-145, potently activated T-cells and enhanced T-cell priming, differentiation, and memory recall responses. In vivo, the antitumor activity of MCLA-145 was superior to immune checkpoint inhibitor comparators and was related to intra-tumor recruitment and expansion of CD8+ T-cells. No graft-versus-host disease was detected, unlike other Abs that inhibit the programmed death (PD)-1 and PD-L1 pathways. MCLA-145 is currently being evaluated in an open label, single-agent dose-escalation study with expansion cohorts for confirmation of dose/safety and preliminary efficacy in advanced or metastatic malignancies (NCT03922204).

Some of these novel bsAbs are reported in Table 3. However, to date, efficacy, and safety results are still pending. 

## 4. Conclusions

Introduction of new target and immune therapies is rapidly changing the landscape in hematology. Indeed, the discovery that the immune system can be an effective cell-specific armament has provided a new therapeutic option. Since the introduction of blinatumomab for R/R B-ALL, there have been significant efforts in the development of other bsAbs across the spectrum of iNHLs and aNHLs. In recent years, advances in CAR T-cell strategies have briefly eclipsed the ongoing development of bsAbs. However, the potential efficacy of bsAbs in CAR T-cell failures have supported CAR T-cell and bsAb-mediated therapeutic approaches, rekindling interest in bsAbs development [1]. 

A central challenge is the production of novel bsAbs with high-quality and limited or negligible side effects. 

Advanced protein and manufacturing engineering technologies in the Ab field have stimulated the development of bsAbs and their derivatives, which represent one of the fastest growing new generations of Ab therapies. Variations in the structure design of bsAbs either in the variable fragments or IgG-like formats or using a combination of both could represent future scenarios. 

Currently, a variety of bsAbs are under development, with encouraging data. However, to date, long-term efficacy and safety results are still pending. 

Moving forward, continued efforts to improve their industrial-scale design, production, and purification will make bsAbs an increasingly robust therapeutic option. Their full value is yet to be defined and will be one of the most important tests of conventional therapy in the next decade.

## Figures and Tables

**Table 1:** Summary of principal reported trials.

	Trial	Enrolled Patients	ORR	PFS	CRS	ICANS-Like
Blinatumomab	Phase 1 [42]	r/r NHL (*N* = 38); aNHL (*N* = 5)	64% (CR 36%)	Median PFS 1.5 years (median follow-up 4.6 years)	20% > G3	22% G 3
	Phase 2 [43]	r/r aNHL (*N* = 25)	43% (CR19%)	Median PFS 3.7 years (median follow-up 15 months)	13% > G3	22% G 3
Glofitamab	Phase 1 [44]	r/r NHL (*N* = 52); aNHL (*N* = 10)	67% (CR 54%) iNHL/61% (CR 54%) aNHL	NR	4% > G3	NR
Mosunetuzumab	Phase 1/2b [45]	r/r NHL (*N* = 270); aNHL (*N* = 116)	63% (CR 43%) iNHL/37% (CR 19%) aNHL	NR	1% G3; no G4	1.1% G3
Odronetoxomab	Phase 1 [46]	r/r NHL (*N* = 136); aNHL (*N* = 78)	55% (CR 55%)/33% (CR 21%) in CAR T r/r	NR	7% > G3	4% G3
Epcoritamab	Phase 1 [47]	r/r NHL (*N* = 68); aNHL (*N* = 46)	80% (CR 60%) iNHL/91% (CR 55%) aNHL (for maximum dose)	NR	no G3	3% G3

**Table 2:** Ongoing trials (ClinicalTrial.gov, accessed on 15 October 2021).

Drug	Status	Study	Target Population	Treatment	Intervention Model	Number of Partecipants
Blinatumomab	Recruiting	NCT03114865	Acute lymphoblastic leukemia (ALL) and B-cell non-Hodgkin lymphoma (NHLs)	Blinatumomab	Open label, phase 1b/2 study	64
Blinatumomab	Recruiting	NCT02568553	Relapsed non-Hodgkin lymphoma	Lenalidomide and blinatumomab	Open label, phase 1 study	44
Blinatumomab	Active, not recruiting	NCT03072771	DLBCL post-ASCT	Blinatumomab	Open label, phase 1 study	14
Blinatumomab	Active, not recruiting	NCT03340766	Relapsed or refractory DLBCL	Blinatumomab in combination with pembrolizumab	Phase 1b open label study	31
Glofitamab	Recruiting	NCT04914741	Younger, higher-risk patients with diffuse large B cell lymphoma	Combination of glofitamab and R-CHOP or pola-RCHP	Open label, multi-centre, phase 1b/2, parallel arm study	80
Glofitamab	Recruiting	NCT04408638	Relapsed/refractory diffuse large B-cell lymphoma	Glofitamab in combination with gemcitabine + pxaliplatin	Phase III, open label, multicenter, randomized study	270
Glofitamab	Recruiting	NCT03467373	Relapsed/refractory NHLs and untreated diffuse large B-cell lymphoma	Glofitamab in combination with rituximab or pbinutuzumab plus CHOP	Phase 1B, multi-center, dose-finding study	172
Glofitamab	Recruiting	NCT03075696	Relapsed/refractory B-cell non-Hodgkin’s lymphoma	Glofitamab as a single agent and in combination with obinutuzumab	Phase 1b/2, multicenter, open label, dose-escalation study	860
Glofitamab	Recruiting	NCT04077723	Relapsed/refractory B-cell non-Hodgkin’s lymphoma	Combination with obinutuzumab and glofitamab	Phase 1b/2, open label, dose-escalation study	362
Glofitamab	Recruiting	NCT03533283	Relapsed/refractory B-cell non-Hodgkin’s lymphoma	Glofitamab and atezolizumab or polatuzumab vedotin	Open label, single arm, multicenter, dose finding, phase 1b study	140
Glofitamab	Recruiting	NCT04657302	Relapsed/refractory diffuse large B-cell lymphoma	Glofitamab as single agent	Phase I, open label, multicenter study	30
Glofitamab	Recruiting	NCT04980222	Untreated diffuse large B-cell lymphoma	Glofitamab in combination with rituximab plus CHOP	Phase II, open label, multicenter study	40
Glofitamab	Recruiting	NCT04889716	Relapsed or refractory diffuse large B-cell or transformed follicular lymphomas	Glofitamab or mosunetuzumab after CAR T-cells	Open label, phase 2 study	42
Glofitamab	Recruiting	NCT04703686	Relapse/refractory lymphomas	Glofitamab after CAR T-cell therapy	Open label, phase 2 study	78
Glofitamab	Active, not recruiting	NCT04313608	Relapsed or refractory diffuse large B-cell lymphoma and high-grade large B-cell lymphoma	Glofitamab or mosunetuzumab in combination with gemcitabine plus oxaliplatin	Phase 1b, open label, multicenter study	20
Epcoritamab	Recruiting	NCT04628494	Relapsed/refractory diffuse large B-cell lymphoma	Epcoritamab	Randomized, open label, phase 3 trial	480
Epcoritamab	Recruiting	NCT04663347	B-cell non-Hodgkin lymphoma	Epcoritamab in combination with other standard of care	Phase 1b/2, open label trial	270
Epcoritamab	Recruiting	NCT03625037	Relapsed, progressive, or refractory B-Cell lymphoma	Epcoritamab GEN3013 (DuoBody^®^-CD3xCD20)	Phase 1/2, open label safety trial	486
Epcoritamab	Recruiting	NCT04542824	Relapsed, progressive, or refractory B-cell lymphoma (JAPANESE PATIENTS)	Epcoritamab	Phase 1/2, open label, dose-escalation trial	73
Odronextamab	Recruiting	NCT03888105	Relapsed or refractory B-cell non-Hodgkin lymphoma	Odronextamab	Open label, phase 2 study	512
Odronextamab	Recruiting	NCT02290951	B-cell non-Hodgkin lymphoma (NHLs) and chronic lymphocytic leukemia (CLL)	Odronextamab	Open label, multi-center phase 1 study	256
Mosunetuzumab	Recruiting	NCT03671018	B-cell non-Hodgkin lymphoma	Mosunetuzumab in combination with polatuzumab vedotin	Open label, randomized, multicenter, phase 1b/2 study	262
Mosunetuzumab	Recruiting	NCT03677154	Diffuse large B-cell lymphoma following first-line immunochemotherapy or untreated diffuse large B-cell lymphoma	Monotherapy or in combination with polatuzumab vedotin	Phase 1/2 study	188
Mosunetuzumab	Active, not recruiting	NCT04313608	Relapsed or refractory diffuse large B-cell lymphoma, and high-grade large B-cell lymphoma	Mosunetuzumab or glofitamab in combination with gemcitabine plus oxaliplatin	Phase 1b, open label, multicenter Study	20
Mosunetuzumab	Active, not recruiting	NCT03677141	Untreated diffuse large B-cell lymphoma	Mosunetuzumab in combination with CHOP or CHP-polatuzumab vedotin	Phase 1b/2, open label, multicenter, randomized, Controlled study	160
Mosunetuzumab	Not yet recruiting	NCT04792502	Untreated FL or MZL	Mosunetuzumab with lenalidomide augmentation	Phase 2, open label study	52
Mosunetuzumab	Not yet recruiting	NCT04889716	Relapsed or refractory diffuse large B-cell or transformed follicular lymphomas	Mosunetuzumab or glofitamab after CAR T-cells	Phase 2 study	42
Mosunetuzumab	Recruiting	NCT02500407	Relapsed or refractory B-cell NHLs and CLL	Mosunetuzumab as a single agent and combined with atezolizumab	Open label, multicenter, phase 1/2 study	836

**Table 3:** Novel bsAbs (ClinicalTrial.gov, accessed on 15 October 2021).

Name	Target	Population	Phase	Treatment	NR Patients	Clinicaltrials.Gov Identifier
MCLA-145	PD-L1/CD137	r/r B-cell lymphoma	1	Dose escalation	118	NCT03922204
TG-1801	CD47/CD19	r/r B-cell lymphoma	1b	Alone or in combination with ublituximab	60	NCT03804996
TNB-486	CD19/CD3	r/r B-cell lymphoma	1	Dose escalation	80	NCT04594642
MT103	CD19/CD3	r/r B-cell lymphoma	1	Dose escalation/single agent	76	NCT00274742
IMM0306	CD20/CD3	r/r B-cell lymphoma	1	Dose escalation	90	NCT04746131
AK104	PD-1/CTLA-4	r/r peripheral T-cell lymphoma	1b/2	Dose escalation/single agent	80	NCT04444141
IBI318	anti-PD1/PD-L1	r/r extranodal NK/T-cell lymphoma	1b/2	Dose escalation/single agent	129	NCT04602065
JNJ-75348780	CD22/CD3	r/r B-cell lymphoma	1	Dose escalation	120	NCT04540796
NVG-111	ROR1/CD3	CLL/SLL and MCL	1b/2	Dose escalation	90	NCT04763083
GB261	CD20/CD3	r/r B-cell lymphoma	1b/2	Dose escalation/single agent	460	NCT04923048
REGN1979	CD20/CD3	r/r B-cell lymphoma	1	Dose escalation/single agent	172	NCT02651662

## Data Availability

Data sharing not applicable.
